# Three Immune-Related Prognostic mRNAs as Therapeutic Targets for Pancreatic Cancer

**DOI:** 10.3389/fmed.2021.649326

**Published:** 2021-04-01

**Authors:** Cangang Zhang, Yueji Zou, Yanan Zhu, Yi Liu, Hui Feng, Fan Niu, Pengcheng He, Haibo Liu

**Affiliations:** ^1^Department of Pathogenic Microbiology and Immunology, School of Basic Medical Sciences, Xi'an Jiaotong University, Xi'an, China; ^2^Department of Imaging, Penglai Traditional Chinese Medicine Hospital, Penglai, China; ^3^Jiangsu ALF Biotechnology Co., Ltd., Nanjing, China; ^4^Department of Hematology, The First Affiliated Hospital of Xi'an Jiaotong University, Xi'an, China

**Keywords:** pancreatic cancer, met, OAS1, OASL, therapeutic targets, prognosis

## Abstract

**Objective:** Pancreatic cancer is a highly lethal malignancy globally. This study aimed to probe and validate immune-related prognostic mRNAs as therapeutic targets for pancreatic cancer.

**Methods:** Gene transcriptome data of pancreatic cancer and normal pancreas were retrieved from TCGA-GTEx projects. Two thousand four hundred and ninety-eight immune-related genes were obtained from the IMMUPORT database. Abnormally expressed immune-related genes were then identified. Under univariate and multivariate cox models, a gene signature was constructed. Its predictive efficacy was assessed *via* ROCs. The interactions between the 21 genes were analyzed by Spearson analysis and PPI network. Using the GEPIA and The Human Protein Atlas databases, their expression and prognostic value were evaluated. The TIMER database was utilized to determine the relationships between MET, OAS1, and OASL mRNAs and immune infiltrates. Finally, their mRNA expression was externally verified in the GSE15471 and GSE62452 datasets.

**Results:** An immune-related 21-gene signature was developed for predicting patients' prognosis. Following verification, this signature exhibited the well predictive performance. There were physical and functional interactions between them. MET, OAS1, and OASL mRNAs were all up-regulated in pancreatic cancer and associated with unfavorable prognosis. They showed strong correlations with tumor progression. Furthermore, the three mRNAs were distinctly associated with immune infiltrates. Their up-regulation was confirmed in the two external datasets.

**Conclusion:** These findings identified three immune-related prognostic mRNAs MET, OAS1, and OASL, which may assist clinicians to choose targets for immunotherapy and make personalized treatment strategy for pancreatic cancer patients.

## Introduction

Pancreatic cancer is a highly lethal disease with fairly high mortality globally (1). Annual mortality is nearly equal to incidence in many countries such as China (2). The 5-year survival is ~7% (3). Its unfavorable prognosis is mainly attributed to local infiltration and distant metastases. Pancreatic ductal adenocarcinoma accounts for around 95% of all cases. Only 10% of cases are due to genetic factors (4). Smoking, drinking, and obesity are common modifiable risk factors for this disease (5). Routine therapy methods like surgery offer dissatisfactory clinical outcomes. Only ~20% could benefit from curative surgical resection (6). Recently, immunotherapy has become a promising adjunct treatment for pancreatic cancer (7). Nevertheless, most of pancreatic cancer patients are resistant to most therapies including immunotherapy because tumors may evade immune surveillance (8). Moreover, there is currently no targeted therapy against driver genes in pancreatic cancer. Thus, it is of necessity to develop novel strategies to build up treatment efficacy.

High-throughput sequencing may assist us to probe therapeutic targets for cancer therapies and understand the underlying mechanisms of the anti-cancer efficacy in depth (9). Pancreatic cancer is featured by distinct immune disorders. Components in immune system contribute to the initiation and development of pancreatic cancer (10). Integrated analysis of relationships between immune-related genes and clinical outcomes of pancreatic cancer is critical to explore novel prognostic markers as well as therapeutic targets. For example, Wu et al. found that three immune-related genes CKLF, ERAP2, and EREG showed distinct correlations with pancreatic cancer patients' survival (11). In this study, we identified three immune-related prognostic genes MET, OAS1, and OASL from the 21-gene signature. Following multiple dataset verification, these genes could be promising therapeutic targets as well as prognostic markers in pancreatic cancer.

## Materials and Methods

### Downloaded Datasets

Gene transcriptome data of 165 normal pancreas samples from the Genotype-Tissue Expression (GTEx) project and 178 pancreatic cancer samples from The Cancer Genome Atlas (TCGA) database were downloaded based on the UCSC Xena browser (https://xenabrowser.net/datapages/). Clinical information of these pancreatic cancer patients including gender, pathologic T, N and histologic grade was retrieved from Genomic Data Commons (GDC). Those without available follow-up data were removed. Table 1 listed the clinical features for each patient.

**Table 1:** Clinical features of 178 pancreatic cancer patients from TCGA database.

**Clinical features**	**Number**
**Gender**	
Male	102
Female	83
**Pathologic T**	
T1	7
T2	24
T3	148
T4	4
Tx	1
**Pathologic N**	
N0	50
N1	126
N1b	4
Nx	4
**Histologic grade**	
G1	32
G2	97
G3	51
G4	2
Gx	3

Two microarray datasets GSE15471 and GSE62452 were downloaded from the Gene Expression Omnibus (GEO) database (https://www.ncbi.nlm.nih.gov/gds/). Of which, the GSE15471 dataset included 39 pairs of pancreatic cancer and normal tissues. Meanwhile, the GSE62452 dataset contained 69 pancreatic cancer and adjacent normal tissue specimens (12).

### Screening for Abnormally Expressed Immune-Related Genes

After merging gene matrix of GTEx and TCGA datasets based on the Ensembl IDs, differentially expressed genes (DEGs) between pancreatic cancer and normal pancreas specimens were screened *via* the Linear Models for Microarray Data (limma) package in R (13). The screening criteria were as follows: |log fold change (FC)| > 2 and false discovery rate (FDR) < 0.05. Two thousand four hundred and ninety-eight immune-related genes were obtained from the IMMUPORT database (https://www.immport.org/home). Following integration of DEGs and immune-related genes, abnormally expressed immune-related genes were identified for pancreatic cancer.

### An Immune-Related Gene Signature Construction

For differentially expressed immune-related genes, univariate Cox regression analysis was conducted using TCGA dataset. Genes with *p* < 0.001 were considered related to pancreatic cancer prognosis. Then, candidate genes were screened *via* multivariate Cox regression analysis. Based on them, a risk score was established according to the following formula: risk score = β_1_x_1_ + β_2_x_2_ + … + β_i_x_i_ (where βi indicates the coefficient of gene i, and x_i_ indicates the expression level of gene i). The risk score of each patient was calculated and all patients were separated into high- and low-risk groups in accordance with the median value. Kaplan–Meier survival analysis was conducted between high- and low-risk groups through the survival package in R. The Receiver Operating Characteristic curves (ROCs) for overall survival were drawn utilizing the survivalROC package in R (14). Areas under the curves (AUCs) were calculated for detection of the efficacy to predict survival for the signature and other clinical features (age, gender, grade, pathologic T, pathologic N, and stage). Univariate and multivariate cox regression analyses were utilized to assess whether risk score could be independently predictive of patients' survival. Hazard ratio (HR), 95% confidence interval (CI) and p were calculated. HR > 1 indicated risk factors and HR < 1 indicated protective factors.

### Functional Annotation Analysis

Gene ontology (GO) including biological processes (BP), molecular functions (MF) and cellular components (CC) and Kyoto Encyclopedia of Genes and Genomes (KEGG) functional annotation analyses of the survival-related genes were presented for exploring underlying biological functions *via* the clusterProfiler package in R (15). Terms with adjusted *p* < 0.05 were significantly enriched.

### Protein-Protein Interaction (PPI) Analysis

The physical and functional interactions of proteins from the immune-related gene signature were analyzed *via* the Search Tool for the Retrieval of Interacting Genes Database (STRING) database (http://string-db.org/) (16). The degree of nodes was then calculated.

### Gene Expression Profiling Interactive Analysis (GEPIA)

The expression of MET, OAS1, and OASL in pancreatic cancer and normal samples was confirmed using TCGA-GTEx projects *via* the online GEPIA database (http://gepia.cancer-pku.cn/index.html) (17). Furthermore, overall survival (OS) and disease-free survival (DFS) analyses were generated for high and low expression of MET, OAS1, or OASL groups among pancreatic cancer patients.

### Immunohistochemistry and Immunofluorescence

Immunohistochemistry of MET, OAS1, and OASL proteins in pancreatic cancer and normal pancreas specimens was obtained from The Human Protein Atlas (https://www.proteinatlas.org/). Furthermore, their immunofluorescence images were also downloaded.

### Immune Infiltration Analysis

The correlations between expression or copy number (arm-level deletion, diploid / normal, arm-level gain, and high amplification) of MET, OAS1, and OASL and the abundance of immune infiltrates composed of B cell, CD4^+^ T cell, CD8^+^ T cell, neutrophil, macrophage, and dendritic cell were assessed using the TIMER database (https://cistrome.shinyapps.io/timer/ (18)).

### Statistical Analysis

All analyses were presented utilizing R version 3.5.2. Spearman correlation analysis was used to assess the associations between genes from the gene signature. The strengths of correlations were determined as follows: 0–0.39: weak; 0.40–0.59: moderate; 0.60–0.79: strong; 0.80–1.0: very strong. The differences in gene expression between two subgroups were calculated using the Wilcox test. *P* < 0.05 indicated statistical significance.

## Results

### Construction of a Prognostic Immune-Related Gene Signature for Pancreatic Cancer

**Figure 1:**
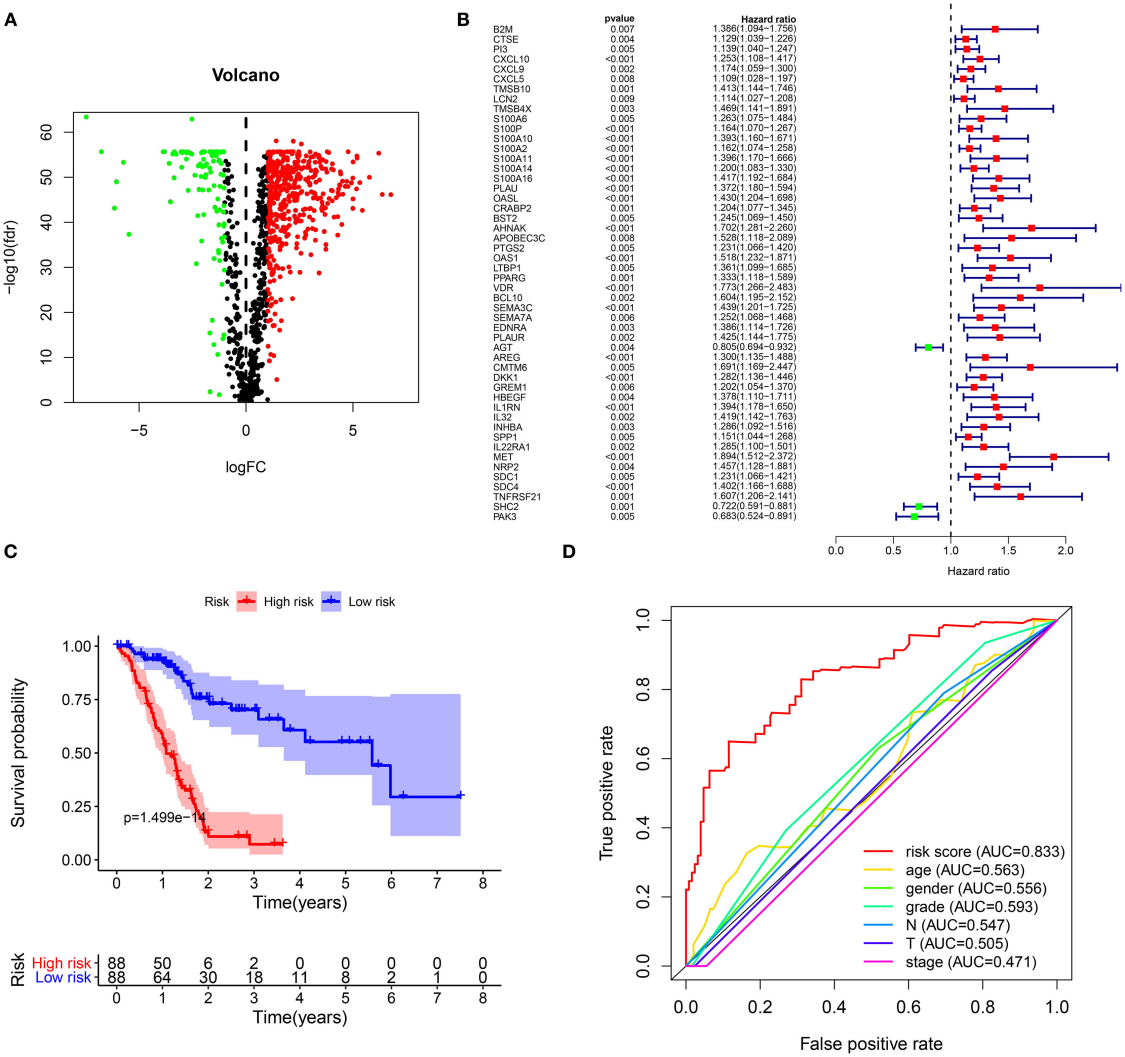
Construction of a prognostic immune-related gene signature for pancreatic cancer. **(A)** Volcano diagram for 192 up- (red) and 37 down-regulated (green) immune-related genes in pancreatic cancer. **(B)** Forest plot for 50 abnormally expressed immune-related genes that were associated with patients' survival. **(C)** Kaplan-Meier curves for overall survival between high (red) and low-risk (blue) groups. **(D)** ROCs of risk score and other clinical prognostic factors for overall survival.

From TCGA-GTEx datasets, 1,737 DEGs with |log FC| > 2 and FDR <0.05 were identified for pancreatic cancer (*n* = 178) than normal pancreas specimens (*n* = 165), which were composed of 962 down- and 775 up-regulated genes listed in Supplementary Table 1. Among all DEGs, 229 were immune-related genes (Figure 1A; Supplementary Table 2). In Figure 1B, 50 abnormally expressed immune-related genes were significantly related to prognosis of pancreatic cancer. Using multivariate Cox regression analysis, 21 candidate genes were identified for constructing a prognostic immune-related gene signature (Table 2). The risk score was calculated for each patient by combining coefficient and expression level. One hundred and seventy-eight patients were separated into high- and low-risk groups in accordance with the median value. Those in the high-risk group exhibited an unfavorable prognosis (*p* = 1.499e-14; Figure 1C). To assess whether the risk score accurately and sensitively predicted patients' survival, ROCs were established. In Figure 1D, the AUC of the risk score for overall survival was 0.833, which was much higher than other clinicopathological factors, suggesting that the risk score possessed high accuracy in predicting survival.

**Table 2:** Multivariate cox regression analysis for 21 candidate genes in pancreatic cancer.

**ID**	**Coefficients**	**Risk ratio**	**95% low**	**95% high**	***P***
B2M	−0.84756	0.42846	0.269333	0.681603	0.000346
PI3	0.126991	1.135407	1.003735	1.284353	0.043462
CXCL9	0.484476	1.623325	1.293371	2.037454	2.93*E*−05
TMSB10	0.359616	1.43278	0.933127	2.199975	0.100254
LCN2	0.178297	1.19518	1.01854	1.402454	0.028882
S100A16	0.873443	2.395142	1.571083	3.651434	4.91*E*−05
OASL	0.511952	1.668545	1.105363	2.518668	0.014819
PTGS2	0.242464	1.274385	1.025728	1.583322	0.028572
OAS1	0.411634	1.509282	0.980257	2.323809	0.061566
LTBP1	0.566777	1.762578	1.172497	2.649628	0.006428
PLAUR	−1.28398	0.276934	0.146236	0.524442	8.11*E*−05
AGT	−0.36056	0.697283	0.545836	0.890749	0.003902
AREG	0.340822	1.406103	1.086432	1.819832	0.0096
CMTM6	−0.64978	0.522162	0.291347	0.935836	0.029056
HBEGF	0.51271	1.669811	1.177601	2.367752	0.004009
SPP1	0.21316	1.237583	1.050946	1.457366	0.010596
IL22RA1	0.170068	1.185385	0.937948	1.498097	0.154535
MET	0.82481	2.281447	1.443826	3.605005	0.00041
NRP2	−0.59908	0.549317	0.341531	0.88352	0.013485
SDC4	−0.62306	0.536299	0.36083	0.797097	0.002059
PAK3	0.669971	1.95418	1.058236	3.608666	0.032287

### The Immune-Related Gene Signature as an Independent Prognostic Factor for Pancreatic Cancer

**Figure 2:**
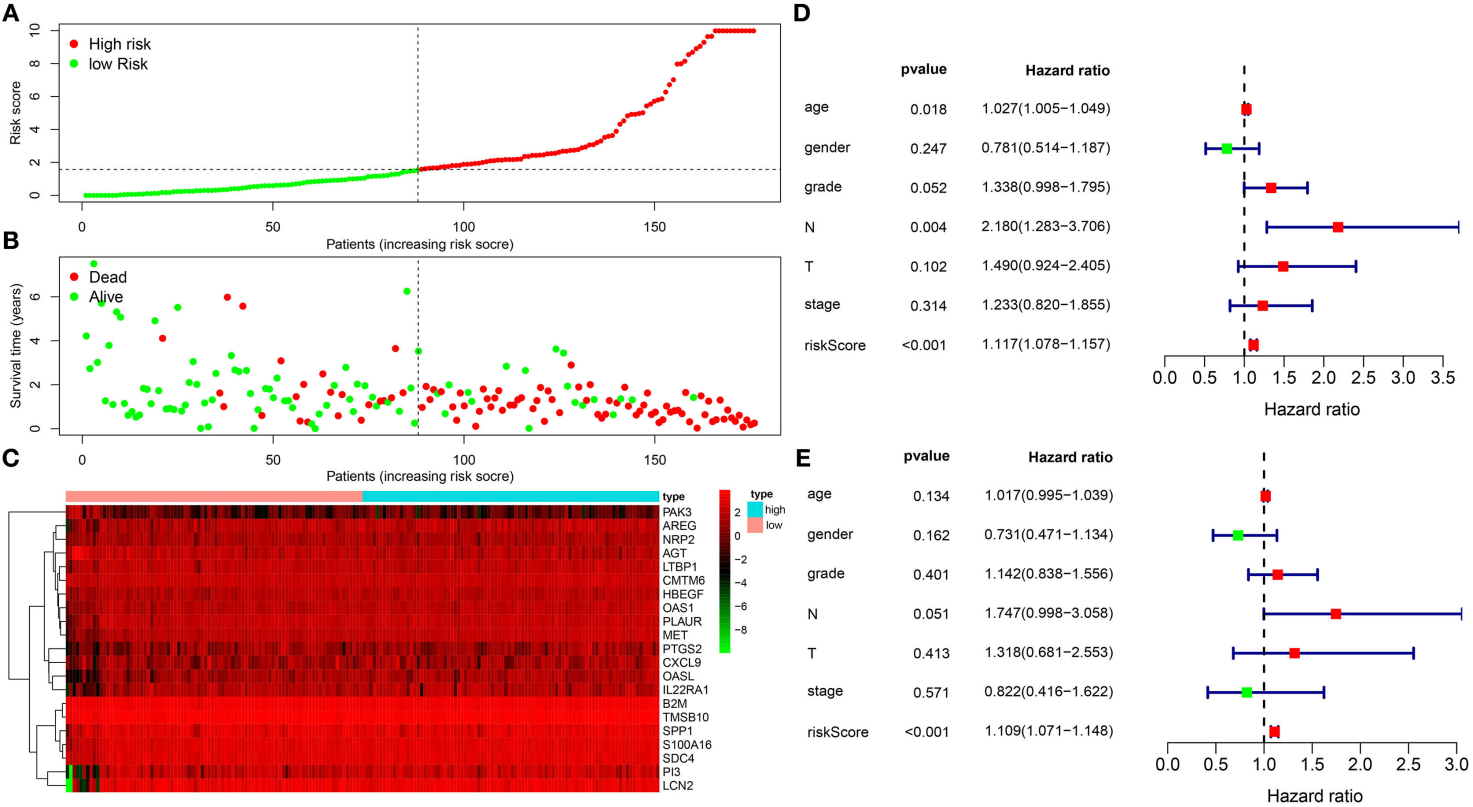
The immune-related gene signature as an independent prognostic factor for pancreatic cancer. **(A)** Risk score ranking. **(B)** Distribution of survival status including dead (red) and alive (green) according to risk scores. **(C)** Heat map for the expression patterns of 21 genes in high and low risk groups. **(D)** Univariate and **(E)** multivariate cox regression analyses for risk score and other clinical indicators.

One hundred and seventy-eight pancreatic cancer patients were ranked according to their risk scores (Figure 2A). As the risk scores elevated, the number of dead patients gradually increased (Figure 2B). Figure 2C depicted the expression patterns of these 21 genes in high and low-risk groups. As shown in univariate cox regression analysis, age [HR (95% CI): 1.027 (1.005–1.049), *p* = 0.018], N [HR (95% CI): 2.180 (1.283–3.706), *p* = 0.004] and risk score [HR (95% CI): 1.117 (1.078–1.157), *p* < 0.001] were risk factors for pancreatic cancer (Figure 2D). Multivariate cox regression analysis revealed that risk score was an independent predictive factor for prognosis of pancreatic cancer (Figure 2E).

### Enrichment Analysis for Survival-Related Genes

**Figure 3:**
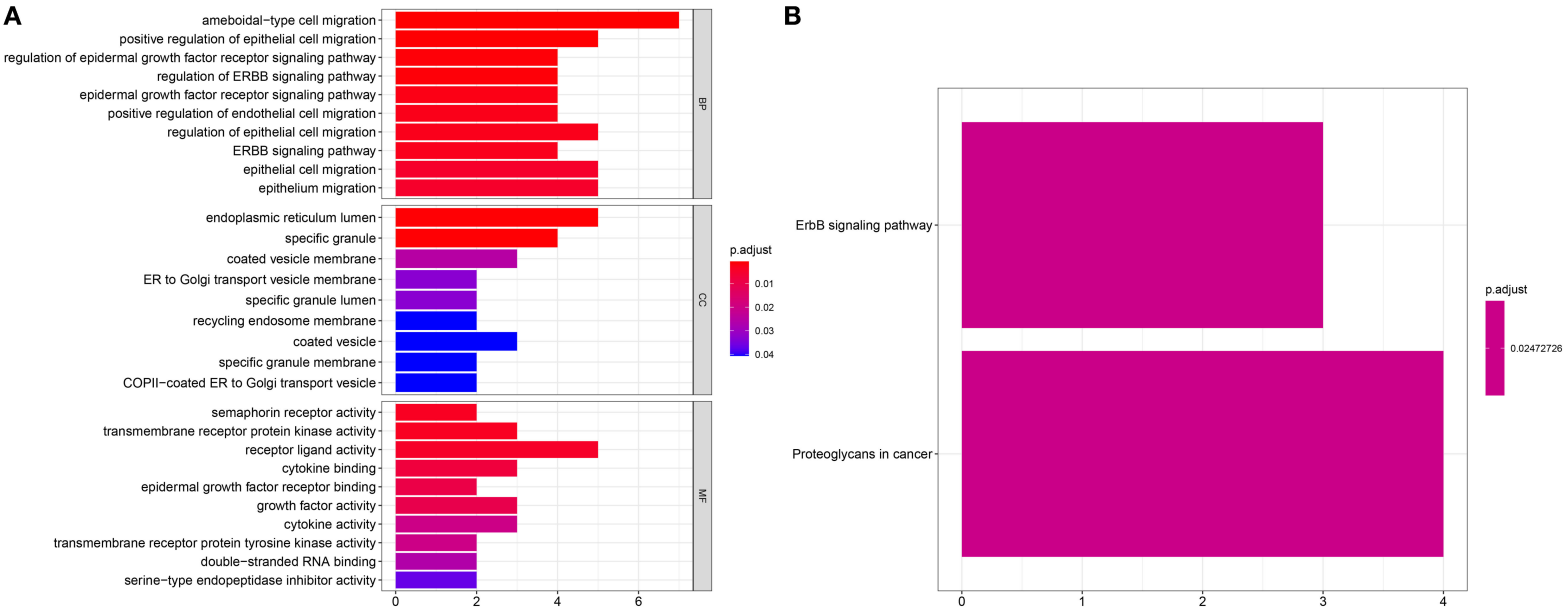
Enrichment analysis for survival-related genes. Bar chart for **(A)** GO functional annotation analysis including biological process (BP), cellular component (CC), and molecular function (MF) and **(B)** KEGG pathway enrichment analysis.

We further probed biological functions of the survival-related genes. In Figure 3A, these genes were distinctly associated with regulation of migration of multiple cells such as epithelial and endothelial cells. Also, they could be involved in key cellular components like endoplasmic reticulum lumen and specific granule and possess different molecular functions such as receptor activity, cytokine binding and growth factor activity. As shown in KEGG enrichment analysis, these genes could participate in ErbB and proteoglycans in cancer pathways (Figure 3B).

### Interactions Between Genes From the Immune-Related Gene Signature

**Figure 4:**
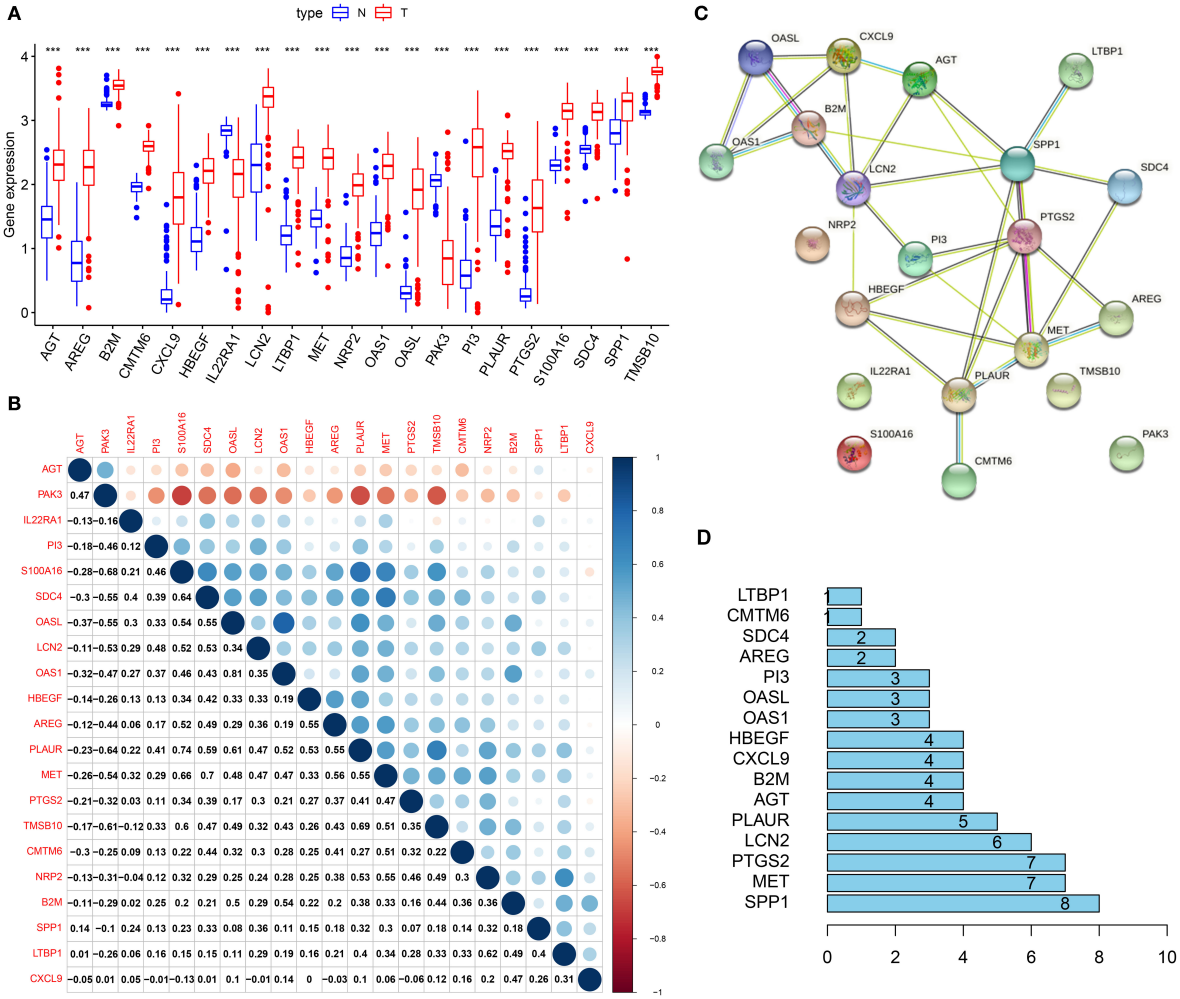
Interactions between genes from the immune-related gene signature. **(A)** Box plot for the expression of 21 genes from the immune-related signature in pancreatic cancer (red) and normal (blue) samples. ***p < 0.001. **(B)** Heat map for the correlations between these genes. **(C)** A PPI network based on these 21 genes. **(D)** The degrees of nodes in the network.

In Figure 4A, genes from immune-related gene signature were all abnormally expressed in pancreatic cancer than normal samples (all *p* < 0.001). Except for IL22RA1 and PAK3, most of them were up-regulated in pancreatic cancer. We further calculated their correlations at the expression levels using Spearson analysis, as shown in Figure 4B. S100A16 was strongly correlated to PAK3 (r = −0.68), SDC4 (r = 0.64), PLAUR (r = 0.74), MET (r = 0.66), and TMSB10 (r = 0.6). PAK3 had strong correlations with PLAUR (r = −0.64) and TMSB10 (r = −0.61). SDC4 exhibited a strong association with MET (r = 0.7). OASL was very strongly associated with OAS1 (r = 0.81). PLAUR showed a strong relationship with TMSB10 (r = 0.69). The PPI network confirmed the closely interactions between them (Figure 4C). Figure 4D listed the degree of each node in the network. We found that SSP1 had the highest degree.

### Up-Regulation of MET, OAS1, and OASL Is Associated With Poor Clinical Outcomes of Pancreatic Cancer

**Figure 5:**
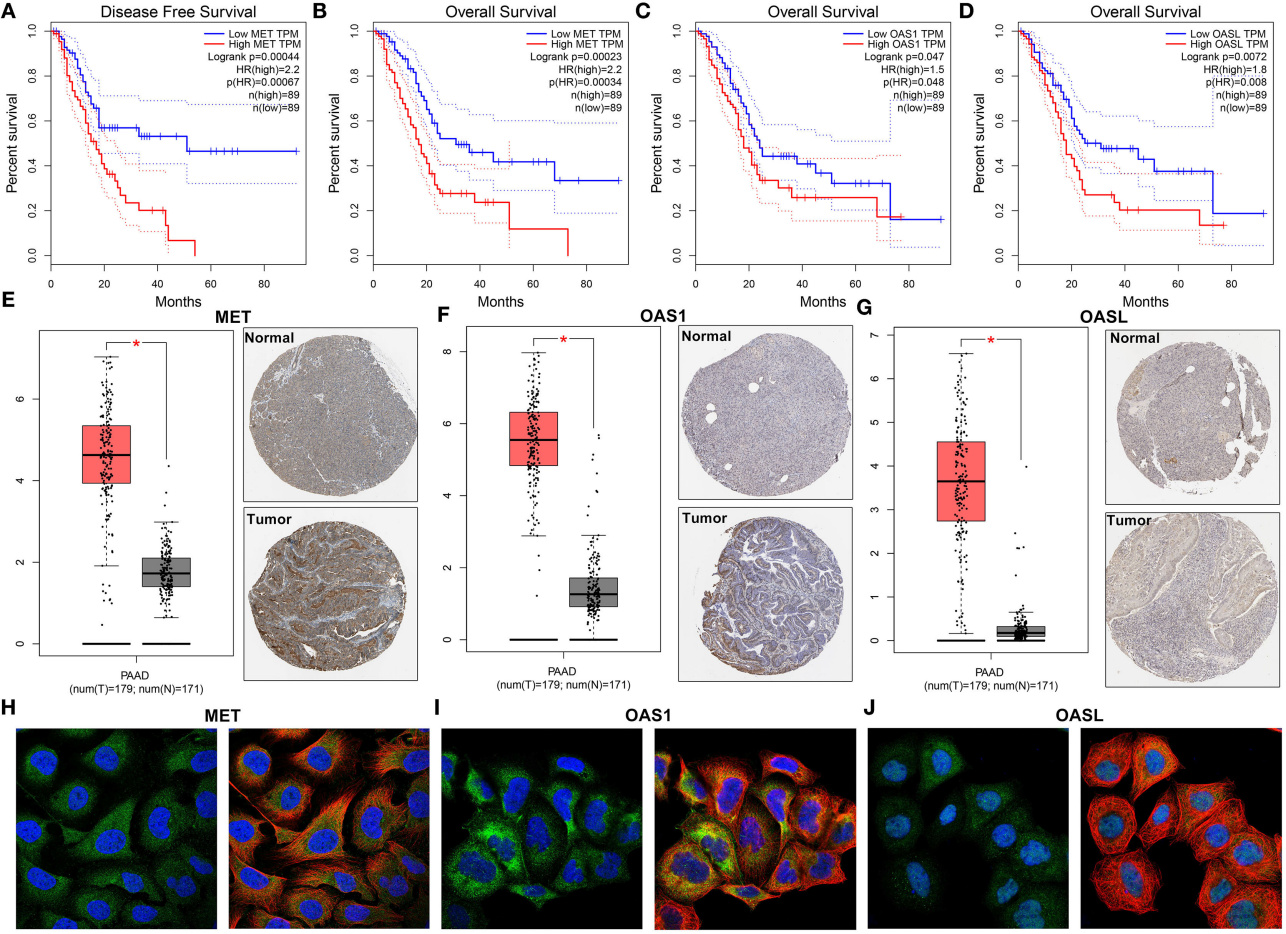
MET, OAS1, and OASL are up-regulated in pancreatic cancer and associated with poor clinical outcomes. Kaplan-Meier curves for **(A)** DFS and **(B)** OS of MET, **(C)** OS of OAS1, and **(D)** OASL *via* the GEPIA. Up-regulation of **(E)** MET, **(F)** OAS1, and **(G)** OASL in pancreatic cancer specimens. The left picture shows the mRNA expression levels from the GEPIA and the right one shows the immunohistochemistry images from The Human Protein Atlas. Immunofluorescence images of **(H)** MET, **(I)** OAS1, and **(J)** OASL in cells. The nucleus was stained blue, the microtubules were stained red, and the MET OAS1 and OASL proteins were stained green. *P<0.05.

**Figure 6:**
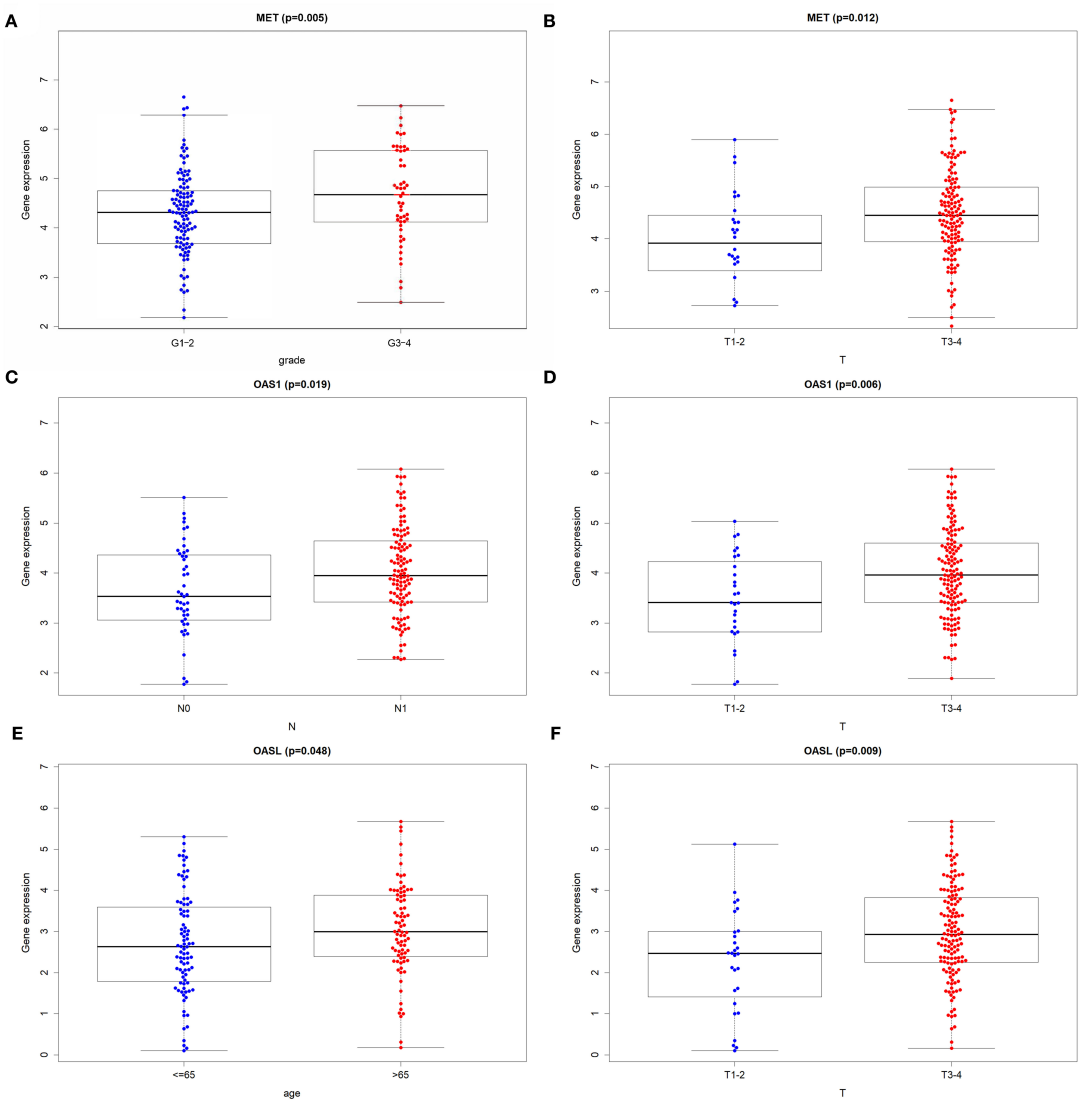
Correlation of MET, OAS1, and OASL expression with clinical features. Box plots for correlation of **(A)** MET with grade and **(B)** pathologic T, correlation of **(C)** OAS1 with pathologic N and **(D)** pathologic T and correlation of **(E)** OASL with age and **(F)** pathologic T.

Among 21 genes from the immune-related signature, MET, OAS1, and OASL were significantly associated with prognosis of pancreatic cancer patients. Patients with high MET expression were indicative of shorter DFS (*p* = 0.00044; Figure 5A) and OS (*p* = 0.00023; Figure 5B) time than those with low expression. Furthermore, high OAS1 (*p* = 0.047; Figure 5C) and OASL (*p* = 0.0072; Figure 5D) expression was distinctly related to poorer OS. MET (Figure 5E), OAS1 (Figure 5F), and OASL (Figure 5G) were all up-regulated at the mRNA and protein levels. Immunofluorescence results demonstrated that MET (Figure 5H), OAS1 (Figure 5I), and OASL (Figure 5J) were mainly distributed in cytoplasm and nucleus. This study further assessed whether MET, OAS1, and OASL expression was in association with clinical features. The data showed that MET expression was significantly higher in G3-G4 (*p* = 0.005; Figure 6A) and T3-T4 (*p* = 0.012; Figure 6B). Furthermore, higher OAS1 expression was detected in N1 (*p* = 0.019; Figure 6C) and T3-T4 (*p* = 0.006; Figure 6D). There was higher OASL expression in >65 (*p* = 0.048; Figure 6E) or T3-T4 (*p* = 0.009; Figure 6F) patients. These findings indicated that MET, OAS1, and OASL might be related to pancreatic cancer progression.

### MET, OAS1, and OASL Correlates With Immune Infiltrates

**Figure 7:**
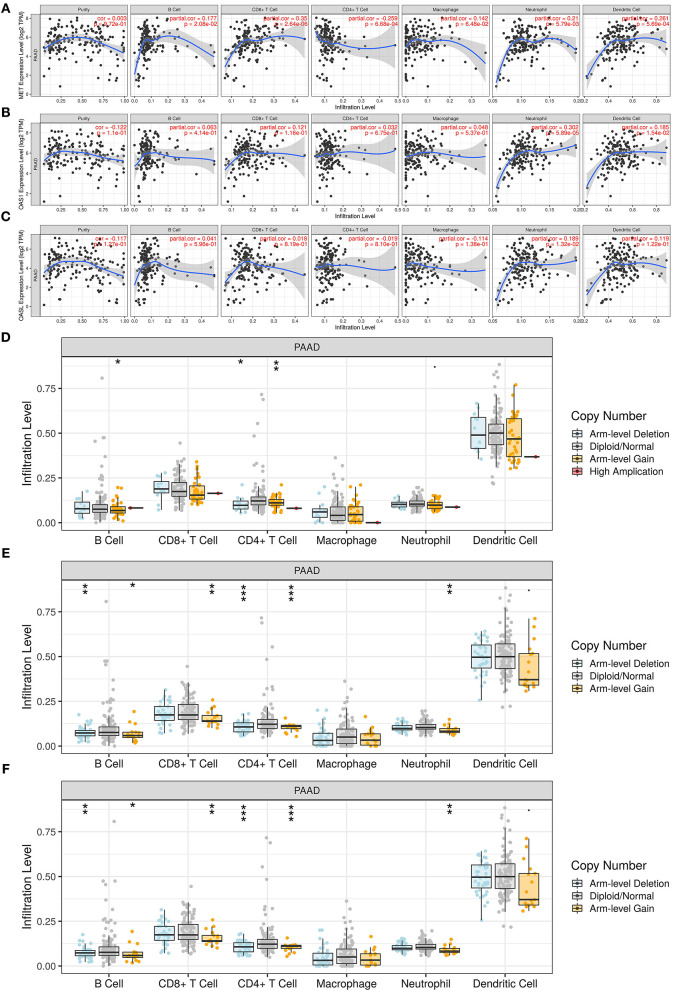
Association of MET, OAS1, and OASL with immune infiltrates *via* the TIMER database. **(A–C)** Correlation of **(A)** MET, **(B)** OAS1, and **(C)** OASL expression with immune infiltrates. **(D–F)** Correlation of copy number of **(D)** MET, **(E)** OAS1, and **(F)** OASL with immune infiltrates. *p < 0.05; **p < 0.01; ***p < 0.001.

Utilizing the TIMER database, we analyzed the correlation of MET, OAS1, and OASL expression with immune infiltrates. Our data suggested that MET expression was significantly correlated to B cell (r = 0.177, *p* = 2.08e-02), CD8 + T cell (r = 0.35, *p* = 2.64e-06), CD4 + T cell (r = −0.259, *p* = 6.68e-04), neutrophil (r = 0.21, *p* = 5.79e-03), and dendritic cell (r = 0.261, *p* = 5.69e-04) in Figure 7A. OAS1 expression exhibited significant associations with neutrophil (r = 0.302, *p* = 5.89e-05) and dendritic cell (r = 0.185, *p* = 1.54e-02; Figure 7B). OASL expression showed a significant correlation with neutrophil (r = 0.189, *p* = 1.32e-02; Figure 7C). Moreover, copy number of MET, OAS1, and OASL was also correlated to immune infiltrates. In Figure 7D, there were significant associations between copy number of MET and B cell and CD4 + T cell. Copy number of OAS1 (Figure 7E) and OASL (Figure 7F) was distinctly correlated to B cell, CD4 + T cell, CD4 + T cell, and neutrophil.

### Validation of MET, OAS1, and OASL Expression in External Datasets

**Figure 8:**
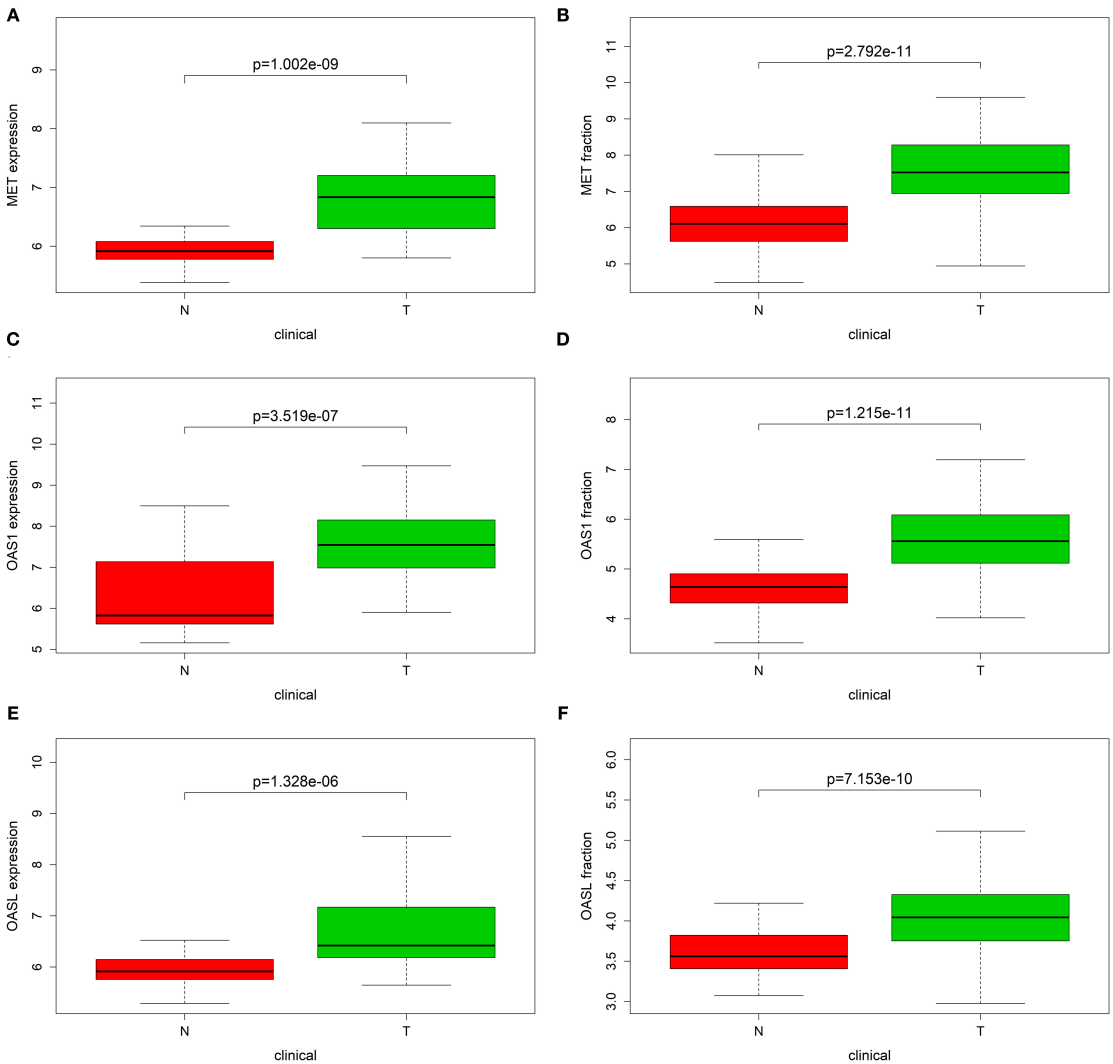
Validation of MET, OAS1, and OASL expression in external datasets. Box plot showing the expression of **(A,B)** MET, **(C,D)** OAS1, and **(E,F)** OASL expression in tumor (T) and normal (N) tissue specimens in GSE15471 and GSE62452 datasets.

The expression of MET, OAS1, and OASL in pancreatic cancer was verified in the GSE15471 and GSE62452 datasets. Consistently, MET was up-regulated in pancreatic cancer both in the GSE15471 (*p* = 1.002e-09; Figure 8A) and GSE62452 datasets (*p* = 2.792e-11; Figure 8B). Higher OAS1 expression was detected in pancreatic cancer than para-carcinoma tissues in the GSE15471 (*p* = 3.519e-07; Figure 8C) and GSE62452 datasets (*p* = 1.215e-11; Figure 8D). Meanwhile, OASL expression was markedly elevated in tumor specimens in the GSE15471 (*p* = 1.328e-05; Figure 8E) and GSE62452 datasets (*p* = 7.153e-10; Figure 8F).

## Discussion

This study constructed an immune-related gene signature for predicting clinical outcomes of pancreatic cancer patients. We identified three key genes (MET, OAS1, and OASL) that were all up-regulated in pancreatic cancer and indicated an unfavorable prognosis. Following multiple dataset verification, the three genes might be promising therapeutic targets, which were worthy of further research.

Despite the TNM stage system as an efficient tool to detect tumor stage, there is discrepancy in prognosis based on TNM stage (19). Thus, the efficacy of TNM stage is limited. Gene-based markers have been widely explored for pancreatic cancer in recent years (20). Recently, several prognosis-related gene signatures have been established for pancreatic cancer (21–23). For example, Zhuang et al. developed a prognosis-related lncRNA signature for pancreatic cancer (24). Following comparing other clinical risk factors, the signature exhibited better predictability. Wu et al. constructed 9-gene signature for prediction of survival time of pancreatic cancer patients (25). There is still a lack of immune-related prognostic models. Herein, the immune-related 21-gene signature could accurately and sensitively predict survival time of pancreatic cancer patients. It performed better than other clinicopathological characteristics like age, gender, grade, N, T, and stage. This signature could be independently predictive of patients' prognosis.

The molecular mechanisms of highly aggressive behaviors remain unknown. We analyzed biological functions of survival-related genes. These genes could regulate migration of multiple cells such as epithelial and endothelial cells and were involved in key cellular components like endoplasmic reticulum lumen and specific granule and possess different molecular functions such as receptor activity, cytokine binding, and growth factor activity. These data were indicative that these genes were involved in tumor progression. For example, CCN1/Cyr61 secreted by pancreatic cancer cells may promote migration of endothelial cells (26). Nerve growth factors regulate CD133 functions, thereby promoting migration of pancreatic cancer cells (27). Targeting IL-1 and its receptor can prolong survival time in pancreatic cancer (28). We also found that these genes could participate in ErbB and proteoglycans in cancer pathways. It has been confirmed that dysregulated ErbB signaling promotes tumorigenesis for pancreatic cancer (29). Hence, these survival-related genes might participate in pancreatic tumor progression.

Our study revealed that there were physical and functional interactions between 21 genes from the immune-related gene signature. Among them, MET, OAS1, and OASL were verified to be markedly up-regulated in pancreatic cancer and associated with poor clinical outcomes of patients. Furthermore, MET expression was significantly correlated to infiltration of B cell, CD8 + T cell, CD4 + T cell, neutrophil, and dendritic cell. MET gene is located on chromosome 7q21-31. Changes in MET functions have been a hallmark of multiple cancers including pancreatic cancer (30). MET overexpression induces pancreatic cancer progression (31). Consistently, MET up-regulation was markedly correlated to tumor grade and T stage (32). Dysregulated MET functions correlate with aggressive phenotypes. Consistent with previous research, MET is involved in the crosstalk between tumor cells and tumor microenvironment (30). Thus, targeting MET has been considered as an adjuvant therapy in pancreatic cancer. OAS1 and OASL, 2′-5′-oligoadenylate synthetases, are interferon-induced antiviral enzymes. We found that OAS1 expression exhibited significant associations with neutrophil and dendritic cell infiltration. OASL expression showed a significant correlation with neutrophil infiltration. Consistently, Zhang et al. also found that OAS1 and OASL were correlated to neutrophil cell infiltration in breast cancer (33). Thus, MET, OAS1, and OASL were distinctly correlated to tumor immune microenvironment.

Collectively, we identified three immune-related prognostic genes MET, OAS1, and OASL, which could be promising therapeutic targets as well as prognostic markers for pancreatic cancer. However, several limitations of this study should be pointed out. First, the biological functions of MET, OAS1, and OASL such as their interactions with immune cells in tumor microenvironment need to be explored *in vitro* experiments. Second, their prognostic values should be verified in prospective research.

## Conclusion

In this study, we established an immune-related 21-gene signature for prediction of pancreatic cancer prognosis. This signature could be accurately and independently predictive of patients' survival. Among these genes, MET, OAS1, and OASL were validated to be up-regulated in pancreatic cancer and associated with unfavorable prognosis of patients. Also, there were closely interactions between them and immune infiltrates. Thus, MET, OAS1, and OASL could be potential therapeutic markers in pancreatic cancer.

## Data Availability Statement

The datasets generated for this study can be found in online repositories. The names of the repository/repositories and accession number(s) can be found in the article/Supplementary Materials.

## Author Contributions

HL conceived and designed the study. CZ, FN, and PH conducted most of the experiments, data analysis, and wrote the manuscript. YZo, YZh, YL, and HF participated in collecting data and helped to draft the manuscript. All authors reviewed and approved the manuscript.

## Conflict of Interest

YZh was employed by the company Jiangsu ALF Biotechnology Co., Ltd. The remaining authors declare that the research was conducted in the absence of any commercial or financial relationships that could be construed as a potential conflict of interest. The Handling Editor declared a shared affiliation, though no other collaboration, with one of the authors CZ.

## Supplementary Material

Supplementary Table 1 One thousand seven hundred and thirty-seven abnormally expressed genes in pancreatic cancer.

Supplementary Table 2 Two hundred and twenty-nine differentially expressed immune-related genes in pancreatic cancer.

## Abbreviations

GTEx: Genotype-Tissue Expression
TCGA: The Cancer Genome Atlas
GDC: Genomic Data Commons
GEO: Gene Expression Omnibus
DEGs: differentially expressed genes
limma: Linear Models for Microarray Data
FC: fold change
FDR: false discovery rate
ROCs: Receiver Operating Characteristic curves
AUCs: Areas under the curves
GO: Gene ontology
BP: biological processes
MF: molecular functions
CC: cellular components
KEGG: Kyoto Encyclopedia of Genes and Genomes
PPI: Protein-protein interaction
STRING: Search Tool for the Retrieval of Interacting Genes Database
GEPIA: Gene Expression Profiling Interactive Analysis
OS: overall survival
DFS: disease-free survival
HR: hazard ratio
CI: confidence interval.
